# Somatic complaints and refrain from buying prescribed medications. Results from a cross-sectional study on people 60 years and older living in Kaunas (Lithuania)

**DOI:** 10.1186/2008-2231-20-78

**Published:** 2012-11-20

**Authors:** Aurima Stankuniene, Mindaugas Stankunas, Joaquim JF Soares, Mark Avery, M Gabriella Melchiorre, Francisco Torres-Gonzalez, Raimondas Radziunas, Algirdas Baranauskas, Jutta Lindert

**Affiliations:** 1 Department of Pharmaceutical Technology and Social Pharmacy, Lithuanian University of Health Sciences, A. Mickeviciaus 9, Kaunas LT 44307, Lithuania; 2 School of Public Health, Griffith University, Gold Coast Campus, Queensland, 4222, Australia; 3 Department of Health Management, Lithuanian University of Health Sciences, A. Mickeviciaus 9, Kaunas, LT 44307, Lithuania; 4 Institution for Health Sciences, Department of Public Health Science, Mid Sweden University, Holmgatan 10, Humlegården, Hus M, 851 70, Sundsvall, Sweden; 5 Scientific Technological Area, Socio Economic Research Centre, Italian National Institute of Health and Science on Aging (INRCA), Via Santa Margherita, 5, 3 piano, Ancona, 60124, Italy; 6 Centro de Investigación Biomedica en Red de Salud Mental CIBERSAM-Granada University, Av Madrid, 11, Granada, PC:18071, Spain; 7 Department of Public Health, Protestant University of Applied Sciences, Paulusweg 6, Ludwigsburg, 71638, Germany; 8 Department of Psychology and Sociology, University of Leipzig, Leipzig, Germany

**Keywords:** Use of medication, Somatic complaints, Self-rated health, Elderly, Accessibility, Non-adherence, Lithuania

## Abstract

**Background:**

The use of medicines by elderly people is a growing area of concern in social pharmacy. A significant proportion of older people do not follow the recommendations from physicians and refrain from buying prescribed medications. The aim of this study is to evaluate associations between self-rated health, somatic complaints and refraining from buying prescribed medications by elderly people.

**Findings:**

Data was collected in a cross-sectional study in 2009. We received 624 completed questionnaires (response rate – 48.9%) from persons aged 60–84 years living in Kaunas (Lithuania). Somatic complaints were measured with the 24 item version of the Giessen Complaint List (GBB-24). Logistic regression (*Enter* model) was used for evaluation of the associations between refraining from buying medications and somatic complaints. These associations were measured using odds ratio (OR) and calculating the 95% confidence interval (CI).

The mean scores in total for the GBB scale and sub-scales (exhaustion, gastrointestinal and cardiovascular) were lowest among respondents who did not refrain from buying prescribed medications (means for GBB-24 scale: 21.04 vs. 24.82; p=0.001). Logistic regression suggests that somatic complaints were associated with a increased risk of refraining from buying prescribed medications (OR=1.35, 95% CI=1.15-1.60).

**Conclusions:**

Somatic complaints were significantly associated with the decision to refrain from buying prescribed medications.

## Findings

### Background

The use of medicines by elderly people is a growing concern in social pharmacy and beyond [1,2]. Increasing prices and proportion of out-pocket payments in purchasing necessary pharmaceuticals leads to situations where some elderly people refrain from buying prescribed medications [3,4]. Reports on underuse of medications provide different results for different countries. A recent study revealed that the incidence of this type of refrain varies from 3% in the Australia, Canada, New Zealand and Netherlands to 9% in the United States [5]. A crucial, but understudied link, in understanding the problem of people refraining from buying prescribed medications is patient’s health status. Patient health status and related conditions (such as costs due ill-health) may influence the decision whether or not to purchase medications. Previous research has found that that main cause of this decision has been financial reasons [5,6]. There is evidence, which shows that sex, age, education level, habitation status, employment status, and economical deprivation affects this type of decision [6]. However, the importance of health status to refrain from purchase has not been the focus of prior research, which has primarily about the evaluation of social-economic factors.

Given this background, the aim of this study was to evaluate associations between somatic complaints and refraining from buying prescribed medications by elderly people.

### Methods

Data for this study was collected during the European project ”Elder abuse: a multinational prevalence study – ABUEL” [7]. Participants in this study consisted of randomly selected women and men from the general population living in Kaunas, the second largest city located in the central part of Lithuania. The population of the city is 321,000 (estimated 2011). The Residents’ Register Service maintained by the Ministry of the Interior provided the study sample of 1,276 individual people. Inclusion criteria were for people who: 1) were age 60–84 years; 2) did not suffer from dementia or other cognitive impairments ^[a]^; 3) had a legal residential status (national citizens or documented migrants); 4) lived in the community or in sheltered houses; 5) could read and write in Lithuanian; and 6) agreed to participate in the study. Recruitment of eligible participants and data collection was performed during April-July, 2009. Data was collected through face-to-face interviews, carried out by trained interviewers. Completed questionnaires were returned to the research team and data was aggregated for analysis. The total number of returned survey questionnaires was 624 (response rate 48.9%). The investigated sample was representative of the elderly population in Kaunas (from 60 to 84 years) with regard to the main demographic characteristics (gender and age). More detailed description of sampling, data collection procedures, and study limitations are described in a separate paper [8].

The participants completed a standardized questionnaire that included various scales and questions ^[b]^ . For this paper, we have used questions related to refrain from buying prescribed medications and somatic complaints.

Self-reported refrain from buying prescribed medications was measured with two questions: “*Have you ever refrained from buying prescribed medication and care”?* (yes/no choice) and “*What were the reasons for not buying prescribed medications and care”?* (multiple-choice).

*Somatic complaints* were measured with the short version of the Giessen Complaint List (GBB-24), which consists of 24 items (graded from 0 – “*not at all*” to 4 – “*very much*”) about various somatic complaints (e.g. physical weakness) [9]. The total possible score is 96 and the items can be divided into 4 sub-scales (exhaustion, gastrointestinal, cardiovascular, and musculoskeletal). High scores correspond to high levels of somatic complaints. Data from a representative sample of the German population as well as studies in medically ill and mentally affected populations have shown good reliability and validity of the GBB-24 [10,11]. Cronbach’s Alpha was 0.910. In this study, the GBB-24 scores were grouped into four groups based on 25, 50, and 75 percentiles. Consequently ‘Group I’ was assigned when GBB-24 score was 0–10; ‘Group II’ = 11–19; ‘Group III’- 20–31; and ‘Group IV’ - 32–96.

The bivariate relation between somatic complaints and decisions to refrain from buying prescribed medications was analyzed with Kruskall-Wallis test. Continuous variables were presented as a mean and median together with a mean’s 95% confidence intervals. We used logistic regression (*Enter* model) for evaluation of the associations between independent and dependent factors. The dependent variables were: 1) refraining from buying prescribed medication; 2) identification of the ‘*financial problems’* as the reason for not purchasing medications; 3) identification of the ‘*the problems disappeared’* as the reason for not purchasing medications. The independent variables were sex, age, education, habitation status (live alone, or with someone else), present employment status (has paid work, or not), financial strain, and level of somatic complaints. The associations was measured using odds ratio (OR) and calculating the 95% confidence interval (CI). The significance level was set at *P* <0.05. Data was analyzed using the Statistical Package for the Social Sciences for Windows Version 13.0 (SPSS for Windows 13).

The Lithuanian State Data Protection Inspectorate and the Kaunas Regional Bioethics Committee granted permission to undertake this study.

### Results

Of the 624 respondents 35.4% (n=221) were males and 64.6% (n=403) females. The distribution of respondents by age was: 23.1% (n=144) aged 60–64 years, 23.5% (n=147) aged 65–69 years, 23.1% (n=144) aged 70–74 years, 19.2% (n=120) aged 75–79 years, and 11.1% (n=69) aged 80–84 years. The mean age of participants was 70.5 (SD=6.64) years. A significant proportion of the respondents (45.2%) had secondary education. Less than one-third (26.0%) had tertiary education and 28.8% primary or lower than primary education. Worries about daily expenses were reported by 72.9% of the respondents (13.9%, always worried; 21.5%, often worried; 37.5%, quite often worried). Only 27.1% reported no worries about daily expenses.

The results showed that 32.7% (n=204) of respondents had refrained from buying prescribed medications. More detailed presentation of causes of refrain and the associations between refrain and socio-economic factors are published in a separate paper [6].

As shown in Table 1, the mean scores in total GBB-24 scale and sub-scales (exhaustion, gastrointestinal and cardiovascular) were lower among respondents who did not refrain from buying prescribed medications.

**Table 1:** Means, medians and means’ 95% confidence intervals of somatic symptoms (sub-scales and total) by decision to refrain from buying prescribed medications

**GBB-24 scale and sub-scales**	**m / M**_**e**_**(95%CI)**		***P*-values**^**a**^
	**Did not refrain**	**Refrained**	
	**(N=420)**	**(N=204)**	
Exhaustion	6.46 / 6	7.55 / 7	
	(6.00–6.93)	(6.90–8.19)	
Gastrointestinal	2.57 / 1	3.43 / 2	
	(2.25-2.89)	(2.93–3.92)	
Cardiovascular	4.58 / 4	5.66 / 5	
	(4.18–4.98)	(5.05–6.27)	
Musculoskeletal	7.42 / 7	8.19 / 8	
	(6.95–7.90)	(7.50–8.87)	
Total GBB-24	21.04 / 18	24.82 / 22	
	(19.66–22.42)	(22.82–26.80)	

*m*– mean and its standard deviation; *Me* – median; *CI* – confidence interval; *n* – number of observed persons; ^a^ - Kruskall-Wallis test.

Financial problems (48.0%) and disappearance of health problems (40.7%) were the most common reasons for refraining. As shown in Table 2, respondents who reported that financial difficulties were the main reasons for not buying prescribed medications reported more somatic complaints than their counterparts. The opposite tendency was identified for the reason “*The problems disappeared*”.

**Table 2:** Means, medians and means’ 95% confidence intervals of somatic symptoms (sub-scales and total) by causes of refrain from buying prescribed medications

**GBB-24 scale and sub-scales**	**m / M**_**e**_**(95%CI)**				***P*-values**^**a**^
	**Financial problems**		**The problems disappeared**		
	**Yes**	**No**	**Yes**	**No**	
	**(n=98)**	**(n=106)**	**(n=83)**	**(n=121)**	
Exhaustion	8.16 / 8	6.98 / 7	6.96 / 7	7.95 / 7	^1^ P=0.108
	(7.18–9.15)	(6.14–7.82)	(6.04–7.89)	(7.06–8.84)	^2^ P=0.225
Gastrointestinal	4.19 / 4	2.72 / 2	2.55 / 2	4.03 / 4	^1^ P=0.001
	(3.46–4.93)	(2.07–3.37)	(1.86–3.25)	(3.36–4.69)	^2^ P=0.003
Cardiovascular	6.61 / 7	4.77 / 4	5.10 / 5	6.04 / 5	^1^ P=0.002
	(5.72–7.51)	(3.96–5.59)	(4.22–5.98)	(5.20–6.88)	^2^ P=0.219
Musculoskeletal	8.84 / 8.5	7.59 / 7	6.80 / 7	9.14 / 9	^1^ P=0.082
	(7.83–9.85)	(6.65–8.52)	(5.96–7.63)	(8.16–10.12)	^2^ P=0.002
Total GBB-24	27.81 / 26.5	22.06 / 20.5	21.41 / 20	27.16 / 26	^1^ P=0.005
	(24.81–30.81)	(19.50–24.61)	(18.81–24.00)	(24.38–29.94)	^2^ P=0.013

*m*– mean and its standard deviation; *Me* – median; *CI* – confidence interval; *n* – number of observed persons; ^a^ - Kruskall-Wallis test; ^1^ – *P* value for *“Financial problems”*; ^2^ – *P* value for *“The problems disappeared”*.

Logistic regression suggest that somatic complaints were associated with refraining from buying prescribed medications (OR=1.35, 95% CI=1.15-1.60.). The “risk” for identification of “financial problems” or “the problems disappeared” as the reason for not buying the prescribed medications was not statistically significant associated with somatic complaints. Odds ratios for every factor are presented in Table 3.

**Table 3:** Logistic regression analysis of the relation between the risk of refrain from buying prescribed medication, for indentifying the “financial problems” or “the problems disappeared” as the reason for not purchasing medications and selected factors

**Factors**	**OR**	**95% CI**	**p**
*Refrain from buying the prescribed medications (Dv)*			
Being male (Iv)	1.00	0.69-1.45	0.993
Age (each age group) (Iv)	0.82	0.70-0.95	0.008
Education (higher level of education) (Iv)	1.02	0.80-1.30	0.903
Living not alone (Iv)	1.04	0.69-1.57	0.865
Is not employed (Iv)	0.73	0.43-1.22	0.226
Daily worries about expenses (each group of more intensive worries) (Iv)	1.02	0.85-1.21	0.856
Somatic complaints (each group of more intensive somatic complaints) (IV)	1.35	1.15-1.60	<0.001
*Financial problems (Dv)*			
Being male (Iv)	1.18	0.59-2.37	0.650
Age (each age group) (Iv)	0.85	0.63-1.31	0.256
Education (higher level of education) (Iv)	0.50	0.32-0.81	0.004
Living not alone (Iv)	0.73	0.33-1.63	0.443
Is not employed (Iv)	1.34	0.50-3.56	0.564
Daily worries about the expenses (each group of more intensive worries) (Iv)	2.86	1.99-4.10	<0.001
Somatic complaints (each group of more intensive somatic complaints) (IV)	1.13	0.83-1.54	0.442
Problems disappeared (Dv)			
Being male (Iv)	1.06	0.56-2.01	0.854
Age (each age group) (Iv)	1.16	0.89-1.51	0.287
Education (higher level of education) (Iv)	1.71	1.11-2.62	0.015
Living not alone (Iv)	1.35	0.66-2.75	0.409
Is not employed (Iv)	0.83	0.33-2.12	0.702
Daily worries about the expenses (each group of more intensive worries) (Iv)	0.67	0.50-0.91	0.009
Somatic complaints (each group of more intensive somatic complaints) (IV)	0.88	0.66-1.17	0.391

*OR* – odds ratio; *CI* – confidence interval; *p* – significance level; *Dv* – dependent variable; *Iv* – independent variable.

### Discussion

Our study revealed that refraining from buying medication was linked to somatic complaints. This indicates that respondents who refrained from buying prescribed medications, due to financial reasons, had more somatic complaints.

As mentioned earlier, we were not able to find any publications or information regarding the link between self-rated health, somatic complaints and refraining from buying prescribed medications. Thus, our findings may be the first to suggest this association. However, it could be that people who have more chronic conditions and more somatic complaints have to spent more resource to purchase medications. A recent study from Austria has revealed that elderly people with a high Charlson comorbidity score had higher “risk” for polypharmacy [12], which subsequently leads to more intensive use of household budget for purchasing pharmaceutical products. Another study from the United States suggests that people aged ≥65 years with diabetes spent an average of 4.1% of their household income on prescribed drugs, whereas those with conditions such as heart failure, angina and ulcers spent between 3.7% and 3.9% [13]. These expenditures levels leads to an understanding that some medications are not purchased in order to save some money. However, there is debate on this issue. A study with community-dwelling veterans revealed that polypharmacy was positively related with higher incomes and health-related beliefs [14].

It also could be the opposite association – refrain from buying prescribed medications causes more somatic complaints. This explanation can be supported by findings from other studies, which argue that inappropriate medications and low adherence to medication regimes can lead to adverse drug events; significant morbidity and mortality; and increased health care costs [15-17]. Moreover, due to financial difficulties, older people may have the necessity to choose between prescribed medications and food, thus refraining, when possible, from buying the former. Conversely, elderly people may sometimes spend less on basic needs such as groceries, in order to buy medications (including at least taking generic products). Poor nutrition may in turn increase somatic complaints (as physical and psychological problems) [18,19].

Another issue could be that decisions to buy or not buy medications are the result of interaction of various factors. Our study has revealed that age was a very strong factor for the decreased risk of refraining from buying prescribed medications. There is evidence showing a clear correlation between age and somatic complaints [20]. The chronic nature of health problems among the oldest people reduce the risk of refraining from buying the prescribed medications and motivate the oldest to follow more precisely physician recommendations and buy the prescribed drugs [21]. Moreover, most of countries offer some compensation systems for purchasing medications in older age [22,23].

This study has limitations, which are described in a separate paper [8]. However, this short communication does not intend to produce generalized evidence rather the objective is to conduct an initial exploration of the relationship between self-rated health, somatic complaints and refraining from buying prescribed medications. Further investigation in this area is needed.

### Conclusion

Results show that 32.7% of respondents had refrained from buying prescribed medications. This decision was significantly associated with somatic complaints. However, more research is needed to explain the link between refrain from buying prescribed medications and somatic complaints.

## Endnotes

^a^Assessed with the Mini-Cog instrument (Borson et al., 2000).

^b^Complete questionnaire is available at http://www.abuel.org.

## Abbreviations

CI: Confidence interval; M: Mean; n: Number of cases; p: Significance level; χ^2^: Chi-square test; OR: Odds ratio; Dv: Dependent variable; Iv: Independent variable.

## Competing interests

This study was funded by was supported by the Executive Agency for Health and Consumers (EAHC) and participating institutions. The study was designed and performed by ABUEL groups in each participating country. None of the authors has any competing interests.

## Authors’ contributions

AS performed the data analysis and drafted and revised the manuscript. MS collected the data, performed data analysis and revised the manuscript. MA, RR, AB contributed in drafting the manuscript. JS, JL, MM, FG participated in the initial study design, data collection and revision of the article. All authors read and approved the final manuscript.
